# Supplementary figures and images for: Multiple sclerosis patients have an altered gut mycobiome and increased fungal to bacterial richness
Source: PLoS One. 2022 Apr 26;17(4):e0264556. doi: 10.1371/journal.pone.0264556 (PMC9041819; doi:10.1371/journal.pone.0264556)

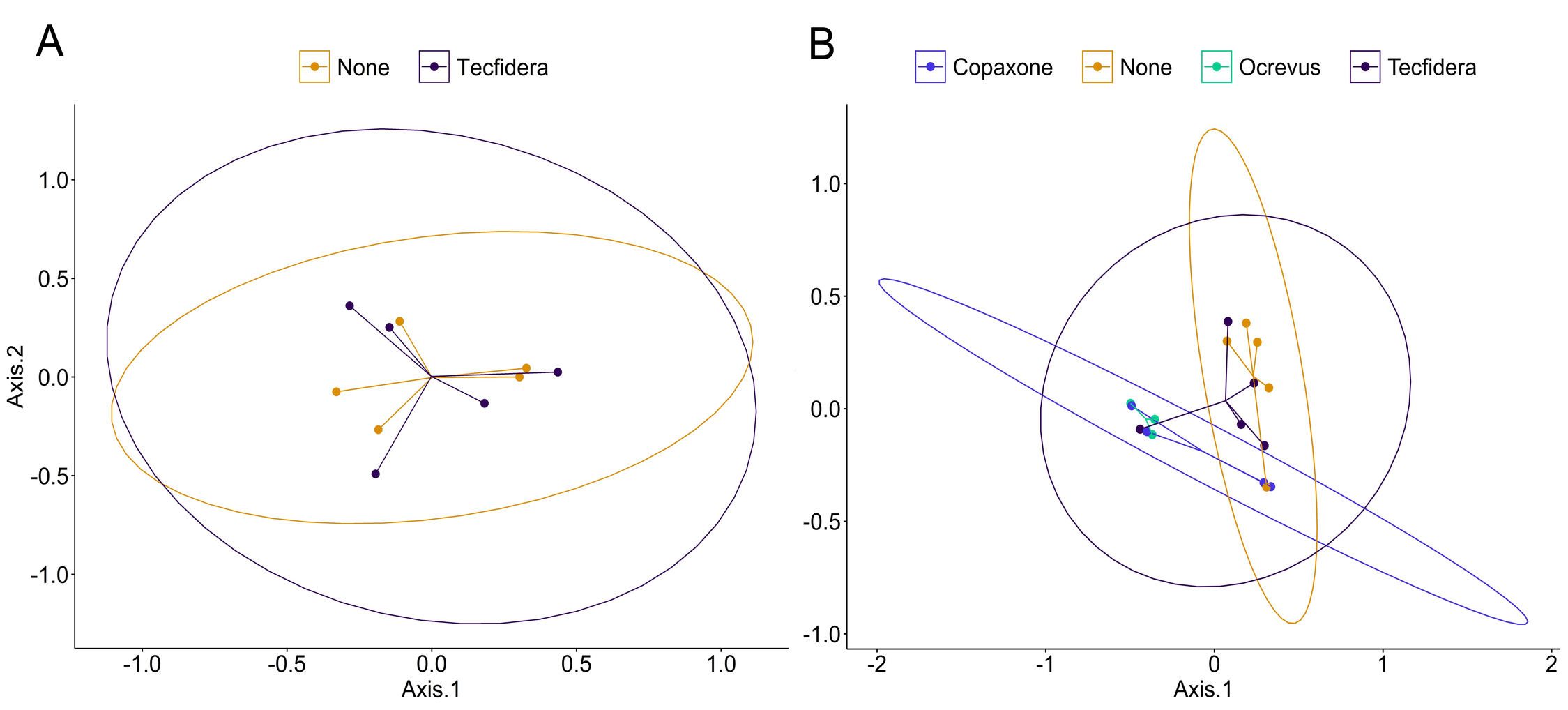

Supplement: S1 Fig — Principal coordinate analysis of beta diversity of the untreated and treated MS groups using Bray-Curtis dissimilarity. (A) No significant effect of treatment on fungal gut composition was demonstrated when comparing MS without treatment to MS treated with dimethyl fumarate (p = 0.872). (B) No significant effect of treatment on fungal gut composition was demonstrated when comparing untreated MS to MS with either dimethyl fumarate, ocrelizumab, or glatiramer acetate (p = 0.163). (TIF) [file pone.0264556.s001.tif]

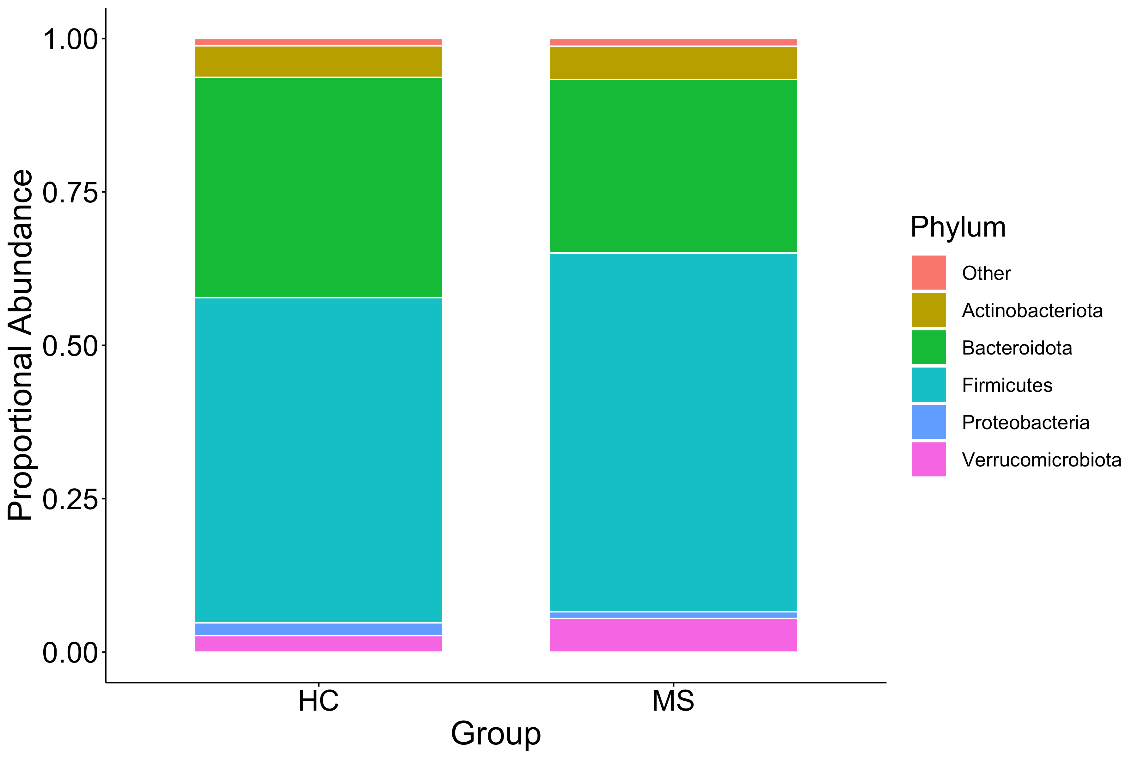

Supplement: S2 Fig — Stacked bar plots representing the proportional abundance of the top 5 bacterial phyla in MS and HC groups. (TIF) [file pone.0264556.s002.tif]

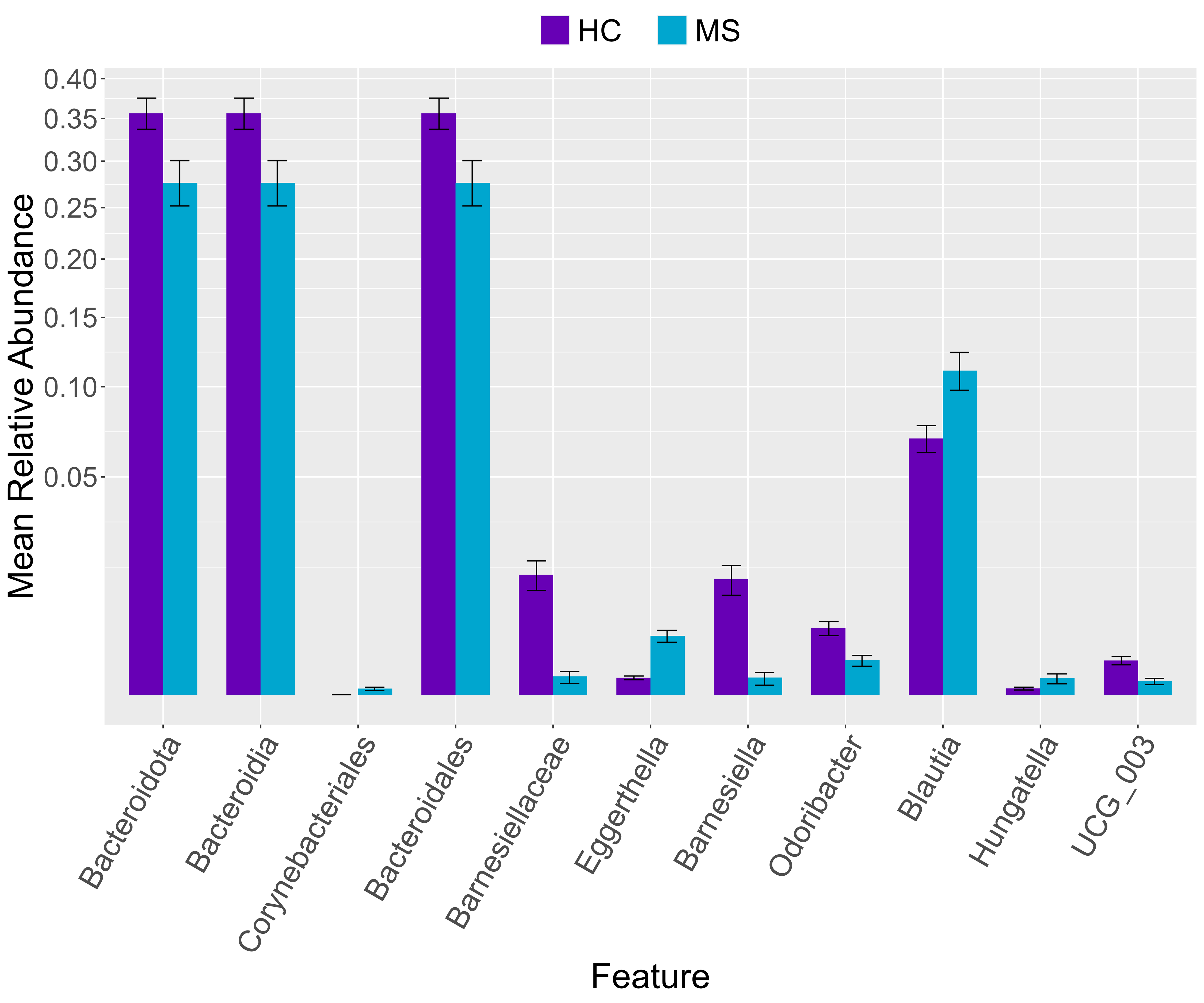

Supplement: S3 Fig — Bar plot showing relative abundances of differentially abundant taxa (p < 0.05) at the family and genus level. (TIF) [file pone.0264556.s003.tif]
