# Supplementary material for: Multiple sclerosis patients have an altered gut mycobiome and increased fungal to bacterial richness
Source: PLoS One. 2022 Apr 26;17(4):e0264556. doi: 10.1371/journal.pone.0264556 (PMC9041819; doi:10.1371/journal.pone.0264556)
Supplement: S1 Table — (DOCX) [file pone.0264556.s004.docx]

Supplemental Table 1: Metadata for bacterial and fungal sequences uploaded to PRJNA732670

| **Bioproject #** | **Biosample #** | **SRR #** | **Sample_name** | **Group** | **Age** | **Sex** | **Treated** | **Treatment** | **BMI** | **Race** |
| --- | --- | --- | --- | --- | --- | --- | --- | --- | --- | --- |
| PRJNA732670 | SAMN19332762 | SRR14660148 | HC14_fungal | HC | 59 | Female | Healthy | Healthy | 20.0 | White |
| PRJNA732670 | SAMN19332762 | SRR14660246 | HC25_fungal | HC | 21 | Male | Healthy | Healthy | 26.8 | White |
| PRJNA732670 | SAMN19332761 | SRR14660161 | MS9_bacterial | MS | 44 | Female | Untreated | None | 40.2 | White |
| PRJNA732670 | SAMN19332762 | SRR14660171 | MS15_fungal | MS | 40 | Female | Treated | Ocrevus | 38.2 | White |
| PRJNA732670 | SAMN19332762 | SRR14660181 | MS6_fungal | MS | 57 | Female | Untreated | None | 27.5 | White |
| PRJNA732670 | SAMN19332761 | SRR14660207 | HC17_bacterial | HC | 30 | Male | Healthy | Healthy | 24.2 | White |
| PRJNA732670 | SAMN19332762 | SRR14660182 | MS5_fungal | MS | 48 | Female | Treated | Copaxone | 24.3 | White |
| PRJNA732670 | SAMN19332762 | SRR14660174 | MS13_fungal | MS | 40 | Female | Treated | Avonex | 19.7 | White |
| PRJNA732670 | SAMN19332761 | SRR14660215 | HC9_bacterial | HC | 28 | Female | Healthy | Healthy | 20.9 | Asian |
| PRJNA732670 | SAMN19332761 | SRR14660250 | MS2_bacterial | MS | 44 | Female | Untreated | None | 20.5 | White |
| PRJNA732670 | SAMN19332761 | SRR14660194 | MS6_bacterial | MS | 57 | Female | Untreated | None | 27.5 | White |
| PRJNA732670 | SAMN19332761 | SRR14660150 | MS10_bacterial | MS | 40 | Female | Treated | Tecfidera | 26.6 | White |
| PRJNA732670 | SAMN19332762 | SRR14660170 | MS16_fungal | MS | 33 | Male | Untreated | None | 34.3 | White |
| PRJNA732670 | SAMN19332762 | SRR14660149 | HC13_fungal | HC | 30 | Female | Healthy | Healthy | 27.2 | Black or African American and White |
| PRJNA732670 | SAMN19332761 | SRR14660198 | HC25_bacterial | HC | 21 | Male | Healthy | Healthy | 26.8 | White |
| PRJNA732670 | SAMN19332762 | SRR14660166 | MS20_fungal | MS | 50 | Female | Treated | Copaxone | 29.5 | White |
| PRJNA732670 | SAMN19332761 | SRR14660217 | HC8_bacterial | HC | 31 | Female | Healthy | Healthy | 25.7 | White |
| PRJNA732670 | SAMN19332761 | SRR14660199 | HC24_bacterial | HC | 57 | Female | Healthy | Healthy | 29.9 | White |
| PRJNA732670 | SAMN19332761 | SRR14660214 | HC10_bacterial | HC | 28 | Female | Healthy | Healthy | 27.0 | White |
| PRJNA732670 | SAMN19332761 | SRR14660172 | MS8_bacterial | MS | 56 | Female | Treated | Avonex | 18.6 | White |
| PRJNA732670 | SAMN19332762 | SRR14660167 | MS19_fungal | MS | 55 | Male | Treated | Ocrevus | 28.4 | White |
| PRJNA732670 | SAMN19332762 | SRR14660248 | HC23_fungal | HC | 52 | Male | Healthy | Healthy | 22.0 | White |
| PRJNA732670 | SAMN19332762 | SRR14660145 | HC17_fungal | HC | 30 | Male | Healthy | Healthy | 24.2 | White |
| PRJNA732670 | SAMN19332762 | SRR14660239 | HC32_fungal | HC | 36 | Female | Healthy | Healthy | 22.0 | White |
| PRJNA732670 | SAMN19332762 | SRR14660165 | MS21_fungal | MS | 27 | Female | Untreated | None | 23.0 | White |
| PRJNA732670 | SAMN19332762 | SRR14660237 | HC33_fungal | HC | 45 | Male | Healthy | Healthy | 23.3 | White |
| PRJNA732670 | SAMN19332762 | SRR14660164 | MS22_fungal | MS | 42 | Female | Treated | Copaxone | 41.3 | White |
| PRJNA732670 | SAMN19332762 | SRR14660158 | HC5_fungal | HC | 25 | Female | Healthy | Healthy | 27.2 | White |
| PRJNA732670 | SAMN19332762 | SRR14660175 | MS12_fungal | MS | 41 | Female | Treated | Tecfidera | 46.1 | White |
| PRJNA732670 | SAMN19332762 | SRR14660142 | HC20_fungal | HC | 63 | Female | Healthy | Healthy | 22.9 | Asian |
| PRJNA732670 | SAMN19332762 | SRR14660236 | HC34_fungal | HC | 58 | Female | Healthy | Healthy | 25.3 | White |
| PRJNA732670 | SAMN19332762 | SRR14660147 | HC15_fungal | HC | 25 | Female | Healthy | Healthy | 20.4 | Asian and White |
| PRJNA732670 | SAMN19332762 | SRR14660140 | HC22_fungal | HC | 22 | Female | Healthy | Healthy | 21.7 | White |
| PRJNA732670 | SAMN19332762 | SRR14660141 | HC21_fungal | HC | 56 | Female | Healthy | Healthy | 28.1 | White |
| PRJNA732670 | SAMN19332762 | SRR14660143 | HC19_fungal | HC | 36 | Female | Healthy | Healthy | 18.2 | White |
| PRJNA732670 | SAMN19332762 | SRR14660144 | HC18_fungal | HC | 60 | Female | Healthy | Healthy | 21.9 | White |
| PRJNA732670 | SAMN19332762 | SRR14660146 | HC16_fungal | HC | 42 | Male | Healthy | Healthy | 24.0 | White |
| PRJNA732670 | SAMN19332762 | SRR14660151 | HC12_fungal | HC | 23 | Female | Healthy | Healthy | 20.4 | White |
| PRJNA732670 | SAMN19332762 | SRR14660152 | HC11_fungal | HC | 58 | Female | Healthy | Healthy | 20.9 | White |
| PRJNA732670 | SAMN19332762 | SRR14660153 | HC10_fungal | HC | 28 | Female | Healthy | Healthy | 27.0 | White |
| PRJNA732670 | SAMN19332762 | SRR14660154 | HC9_fungal | HC | 28 | Female | Healthy | Healthy | 20.9 | Asian |
| PRJNA732670 | SAMN19332762 | SRR14660155 | HC8_fungal | HC | 31 | Female | Healthy | Healthy | 25.7 | White |
| PRJNA732670 | SAMN19332762 | SRR14660156 | HC7_fungal | HC | 63 | Female | Healthy | Healthy | 32.5 | White |
| PRJNA732670 | SAMN19332762 | SRR14660157 | HC6_fungal | HC | 43 | Female | Healthy | Healthy | 30.4 | White |
| PRJNA732670 | SAMN19332762 | SRR14660159 | HC4_fungal | HC | 37 | Female | Healthy | Healthy | 24.4 | White |
| PRJNA732670 | SAMN19332762 | SRR14660160 | HC3_fungal | HC | 38 | Female | Healthy | Healthy | 29.9 | White |
| PRJNA732670 | SAMN19332762 | SRR14660162 | HC2_fungal | HC | 43 | Female | Healthy | Healthy | 24.9 | White |
| PRJNA732670 | SAMN19332762 | SRR14660163 | HC1_fungal | HC | 52 | Male | Healthy | Healthy | 27.3 | White |
| PRJNA732670 | SAMN19332762 | SRR14660168 | MS18_fungal | MS | 52 | Male | Treated | Copaxone | 31.0 | White |
| PRJNA732670 | SAMN19332762 | SRR14660169 | MS17_fungal | MS | 48 | Female | Untreated | None | 32.6 | White |
| PRJNA732670 | SAMN19332762 | SRR14660173 | MS14_fungal | MS | 44 | Female | Treated | Ocrevus | 30.9 | White |
| PRJNA732670 | SAMN19332762 | SRR14660176 | MS11_fungal | MS | 37 | Female | Treated | Tecfidera | 29.5 | White |
| **Bioproject #** | **Biosample #** | **SRR #** | **Sample_name** | **Group** | **Age** | **Sex** | **Treated** | **Treatment** | **BMI** | **Race** |
| PRJNA732670 | SAMN19332762 | SRR14660179 | MS8_fungal | MS | 56 | Female | Treated | Avonex | 18.6 | White |
| PRJNA732670 | SAMN19332762 | SRR14660180 | MS7_fungal | MS | 46 | Female | Treated | Tecfidera | 39.1 | White |
| PRJNA732670 | SAMN19332761 | SRR14660183 | MS7_bacterial | MS | 46 | Female | Treated | Tecfidera | 39.1 | White |
| PRJNA732670 | SAMN19332762 | SRR14660184 | MS4_fungal | MS | 49 | Female | Treated | Tecfidera | 25.7 | White |
| PRJNA732670 | SAMN19332761 | SRR14660216 | MS4_bacterial | MS | 49 | Female | Treated | Tecfidera | 25.7 | White |
| PRJNA732670 | SAMN19332761 | SRR14660218 | HC7_bacterial | HC | 63 | Female | Healthy | Healthy | 32.5 | White |
| PRJNA732670 | SAMN19332761 | SRR14660219 | HC6_bacterial | HC | 43 | Female | Healthy | Healthy | 30.4 | White |
| PRJNA732670 | SAMN19332761 | SRR14660220 | HC5_bacterial | HC | 25 | Female | Healthy | Healthy | 27.2 | White |
| PRJNA732670 | SAMN19332761 | SRR14660221 | HC4_bacterial | HC | 37 | Female | Healthy | Healthy | 24.4 | White |
| PRJNA732670 | SAMN19332761 | SRR14660222 | HC3_bacterial | HC | 38 | Female | Healthy | Healthy | 29.9 | White |
| PRJNA732670 | SAMN19332761 | SRR14660223 | HC2_bacterial | HC | 43 | Female | Healthy | Healthy | 24.9 | White |
| PRJNA732670 | SAMN19332761 | SRR14660224 | HC1_bacterial | HC | 52 | Male | Healthy | Healthy | 27.3 | White |
| PRJNA732670 | SAMN19332761 | SRR14660225 | MS22_bacterial | MS | 42 | Female | Treated | Copaxone | 41.3 | White |
| PRJNA732670 | SAMN19332761 | SRR14660226 | MS21_bacterial | MS | 27 | Female | Untreated | None | 23.0 | White |
| PRJNA732670 | SAMN19332761 | SRR14660227 | MS3_bacterial | MS | 51 | Male | Treated | Tecfidera | 27.1 | White |
| PRJNA732670 | SAMN19332761 | SRR14660228 | MS20_bacterial | MS | 50 | Female | Treated | Copaxone | 29.5 | White |
| PRJNA732670 | SAMN19332762 | SRR14660185 | MS3_fungal | MS | 51 | Male | Treated | Tecfidera | 27.1 | White |
| PRJNA732670 | SAMN19332762 | SRR14660186 | MS2_fungal | MS | 44 | Female | Untreated | None | 20.5 | White |
| PRJNA732670 | SAMN19332762 | SRR14660187 | MS1_fungal | MS | 40 | Male | Treated | Rebif | 22.3 | White |
| PRJNA732670 | SAMN19332761 | SRR14660188 | HC34_bacterial | HC | 58 | Female | Healthy | Healthy | 25.3 | White |
| PRJNA732670 | SAMN19332761 | SRR14660189 | HC33_bacterial | HC | 45 | Male | Healthy | Healthy | 23.3 | White |
| PRJNA732670 | SAMN19332761 | SRR14660190 | HC32_bacterial | HC | 36 | Female | Healthy | Healthy | 22.0 | White |
| PRJNA732670 | SAMN19332761 | SRR14660191 | HC31_bacterial | HC | 46 | Female | Healthy | Healthy | 27.8 | White |
| PRJNA732670 | SAMN19332761 | SRR14660192 | HC30_bacterial | HC | 44 | Female | Healthy | Healthy | 23.2 | White |
| PRJNA732670 | SAMN19332761 | SRR14660193 | HC29_bacterial | HC | 51 | Female | Healthy | Healthy | 22.3 | White |
| PRJNA732670 | SAMN19332761 | SRR14660195 | HC28_bacterial | HC | 61 | Female | Healthy | Healthy | 20.0 | White |
| PRJNA732670 | SAMN19332761 | SRR14660196 | HC27_bacterial | HC | 32 | Female | Healthy | Healthy | NA | White |
| PRJNA732670 | SAMN19332761 | SRR14660197 | HC26_bacterial | HC | 24 | Female | Healthy | Healthy | 19.9 | White |
| PRJNA732670 | SAMN19332761 | SRR14660200 | HC23_bacterial | HC | 52 | Male | Healthy | Healthy | 22.0 | White |
| PRJNA732670 | SAMN19332761 | SRR14660201 | HC22_bacterial | HC | 22 | Female | Healthy | Healthy | 21.7 | White |
| PRJNA732670 | SAMN19332761 | SRR14660202 | HC21_bacterial | HC | 56 | Female | Healthy | Healthy | 28.1 | White |
| PRJNA732670 | SAMN19332761 | SRR14660203 | HC20_bacterial | HC | 63 | Female | Healthy | Healthy | 22.9 | Asian |
| PRJNA732670 | SAMN19332761 | SRR14660204 | HC19_bacterial | HC | 36 | Female | Healthy | Healthy | 18.2 | White |
| PRJNA732670 | SAMN19332761 | SRR14660205 | MS5_bacterial | MS | 48 | Female | Treated | Copaxone | 24.3 | White |
| PRJNA732670 | SAMN19332761 | SRR14660206 | HC18_bacterial | HC | 60 | Female | Healthy | Healthy | 21.9 | White |
| PRJNA732670 | SAMN19332761 | SRR14660208 | HC16_bacterial | HC | 42 | Male | Healthy | Healthy | 24.0 | White |
| PRJNA732670 | SAMN19332761 | SRR14660209 | HC15_bacterial | HC | 25 | Female | Healthy | Healthy | 20.4 | Asian and White |
| PRJNA732670 | SAMN19332761 | SRR14660210 | HC14_bacterial | HC | 59 | Female | Healthy | Healthy | 20.0 | White |
| PRJNA732670 | SAMN19332761 | SRR14660211 | HC13_bacterial | HC | 30 | Female | Healthy | Healthy | 27.2 | Black or African American and White |
| PRJNA732670 | SAMN19332761 | SRR14660212 | HC12_bacterial | HC | 23 | Female | Healthy | Healthy | 20.4 | White |
| PRJNA732670 | SAMN19332761 | SRR14660213 | HC11_bacterial | HC | 58 | Female | Healthy | Healthy | 20.9 | White |
| PRJNA732670 | SAMN19332761 | SRR14660229 | MS19_bacterial | MS | 55 | Male | Treated | Ocrevus | 28.4 | White |
| PRJNA732670 | SAMN19332761 | SRR14660230 | MS18_bacterial | MS | 52 | Male | Treated | Copaxone | 31.0 | White |
| PRJNA732670 | SAMN19332761 | SRR14660231 | MS17_bacterial | MS | 48 | Female | Untreated | None | 32.6 | White |
| PRJNA732670 | SAMN19332761 | SRR14660232 | MS16_bacterial | MS | 33 | Male | Untreated | None | 34.3 | White |
| PRJNA732670 | SAMN19332761 | SRR14660233 | MS15_bacterial | MS | 40 | Female | Treated | Ocrevus | 38.2 | White |
| PRJNA732670 | SAMN19332761 | SRR14660234 | MS14_bacterial | MS | 44 | Female | Treated | Ocrevus | 30.9 | White |
| PRJNA732670 | SAMN19332761 | SRR14660235 | MS13_bacterial | MS | 40 | Female | Treated | Avonex | 19.7 | White |
| PRJNA732670 | SAMN19332761 | SRR14660238 | MS12_bacterial | MS | 41 | Female | Treated | Tecfidera | 46.1 | White |
| PRJNA732670 | SAMN19332762 | SRR14660240 | HC31_fungal | HC | 46 | Female | Healthy | Healthy | 27.8 | White |
| PRJNA732670 | SAMN19332762 | SRR14660241 | HC30_fungal | HC | 44 | Female | Healthy | Healthy | 23.2 | White |
| PRJNA732670 | SAMN19332762 | SRR14660242 | HC29_fungal | HC | 51 | Female | Healthy | Healthy | 22.3 | White |
| PRJNA732670 | SAMN19332762 | SRR14660243 | HC28_fungal | HC | 61 | Female | Healthy | Healthy | 20.0 | White |
| PRJNA732670 | SAMN19332761 | SRR14660251 | MS1_bacterial | MS | 40 | Male | Treated | Rebif | 22.3 | White |
|  |  |  |  |  |  |  |  |  |  |  |
| **Bioproject #** | **Biosample #** | **SRR #** | **Sample_name** | **Group** | **Age** | **Sex** | **Treated** | **Treatment** | **BMI** | **Race** |
| PRJNA732670 | SAMN19332762 | SRR14660244 | HC27_fungal | HC | 32 | Female | Healthy | Healthy | NA | White |
| PRJNA732670 | SAMN19332762 | SRR14660245 | HC26_fungal | HC | 24 | Female | Healthy | Healthy | 19.9 | White |
| PRJNA732670 | SAMN19332762 | SRR14660247 | HC24_fungal | HC | 57 | Female | Healthy | Healthy | 29.9 | White |
| PRJNA732670 | SAMN19332761 | SRR14660249 | MS11_bacterial | MS | 37 | Female | Treated | Tecfidera | 29.5 | White |
